# Complete recovery of deep venous thrombosis from Coombs (+) thrombotic thrombocytopenic purpura: case report

**DOI:** 10.1186/s13019-022-01789-8

**Published:** 2022-03-21

**Authors:** Mi Zhou, Jie Yin

**Affiliations:** 1 Department of Vascular Surgery, Xuanwu Hospital, Capital Medical University, Beijing, China; 2 Department of Cardiology, Shandong Provincial Qianfoshan Hospital, the First Hospital Affiliated With Shandong First Medical University, Jinan, Shandong China; 3 Department of Cardiology, Shandong Province Qianfushan Hospital, Shandong University, Jinan, 250014 China

**Keywords:** Thrombotic thrombocytopenic purpura, Deep vein thrombosis, Plasma exchange

## Abstract

**Background:**

Acute thrombotic thrombocytopenic purpura (TTP) is an aggressive thrombotic microangiopathy that if not treated, can have a 90% mortality rate. Timely, extensive plasma exchange (PEX) has been indicated to reduce the mortality rate to < 10%, but its side effects are not well-known. We present here a case of a patient presented with Comb (+) TTP and developed catheter-associated deep vein thrombosis (DVT).

**Case presentation:**

A 27-year-young man presented with persistent thrombocytopenia and Coombs positive anemia was firstly diagnosed with Evans syndrome. However, he was refractory to a methylprednisolone pulse therapy with a combination of platelet transfusion and eventually developed microangiopathy of central nerve system. Several pathological manifestations of the disease were prevented by PEX. The immediate start of PEX (1500 mL/d) induced a complete remission of acquired TTP and disappearance of neurological signs and symptoms. However, external iliac and femoro-popliteal venous thrombosis was diagnosed subsequently, inferior vena cava filter (IVC) filter was immediately implanted accompanied with anticoagulation therapy. Meanwhile, PEX session was sustained as well as oral anticoagulant (rivaroxaban). 14 days later, the patient got full recovery.

**Conclusions:**

Catheter-related DVT under the setting of TTP should be cautious. It is necessary to start anticoagulation and antiplatelet therapy for thrombosis early, especially in such cases when PLT count > 50 × 10^9^/L.

## Background

Thrombotic thrombocytopenic purpura (TTP) is a rare and potentially fatal hematologic disease. Fifty years ago, before the era of effective treatment, the diagnosis of TTP was based on the progressive appearance of the “pentad” of clinical manifestations: microangiopathic hemolytic anemia, thrombocytopenia, renal and neurological abnormalities, and fever [1]. Recent studies have demonstrated that deficiency in the von Willebrand factor (vWF) cleaving protease ADAMTS13 (a disintegrin and metalloproteinase with thrombospondin motifs 13) causes TTP. The deficiency of ADAMTS13 can be genetic or more common, acquired, resulting from autoimmune production of inhibitory anti-ADAMTS13 antibodies [2].

PEX (plasma exchange) has now become the cornerstone of the management of TTP. Timely, extensive PEX has been indicated to reduce the mortality rate to < 10%, resulting in > 90% short-term effectiveness. However, few attentions have been paid to the complication of PEX since PEX requires insertion of a central venous dialysis catheter, with its risk for hemorrhage, thrombosis and infection [3].

In this case, we describe a young male who was firstly defined by either simultaneous or sequential combination of immune thrombocytopenia and autoimmune hemolytic anemia with a positive direct anti globulin test (DAT) in the absence of known underlying etiology and diagnosed as TTP. He responded well to the timely PEX but suffered catheter-associated thrombosis.

## Case presentation

### Presenting clinical features

A 27-year-old previously healthy male patient (height 176 cm, and weight 79 kg) presented with fever, macrohematuria, and purpura in the lower legs developed 4 days before admission, respectively. He denied drug exposures and recent infectious illness. He had no abdominal pain or diarrhea. Vital signs were normal and physical examination was unremarkable except for petechiae. His mother died of anemia (details unclear). Laboratory findings revealed hemolytic anemia, thrombocytopenia (Table 1), and renal damage (Urinalysis disclosed a proteinuria score of 2+, a red blood cell count of 8.4 per high-power field, a white blood cell count of 4.4 per high-power field, and serum creatinine: normal). Peripheral smear showed numerous schistocytes (1.2%). Prothrombin time, partial thromboplastin time, and renal function test was all within normal limits (Table 2). The laboratory tests showed a direct antiglobulin test (+), indicating peripheral cytopenias, particularly autoimmune cytopenias (AIC) such as autoimmune thrombocytopenia, Anti-SSA, Jo-52 (+). A bone marrow biopsy was also performed, showing only erythroid hyperplasia without other abnormalities. A diagnosis of ES was made given the evidence of immune-mediated hemolysis with thrombocytopenia in the absence of a known etiology, we administered methylprednisolone pulse therapy with the dose of 500 mg/d for 3 consecutive days. At the following days, he had a drop in his Hgb was from 15.2 to 7.4 g/dL, with an elevated LDH level soaring to 4136 U/L.

**Table 1:** Overall laboratory results (Complete blood count and Blood chemistry) of the patient

Component measured	Reference range	Result
Hemoglobin (g/L)	105–140	74
Hematocrit (%)	33–42	22.7
Reticulocyte count (× 10^9^/L)	54	144–336
Total bilirubin (mg/L)	100–1000	66
Indirect bilirubin (mg/L)	0–800	51
Aspartate aminotransferase (U/L)	14–40	50
Lactate dehydrogenase (U/L)	150–500	3489
Haptoglobin (g/L)	0.2–1.9	Undetectable
Platelet count (× 10^9^/L)	100–300	9

**Table 2:** Overall laboratory results (Coagulation function test) of the patient. PT, prothrombin time; APTT, activated partial thromboplastin time

Coagulation function test	Reference range	Result
PT (s)	11–13	12.1
APTT (s)	25–37	37.9
Fibrinogen (g/L)	2–5.5	5.25
Factor Xa activity (%)	85–120	115
Antithrombin III activity (%)	80–120	92

### Neurologic abnormalities

**Fig. 1:**
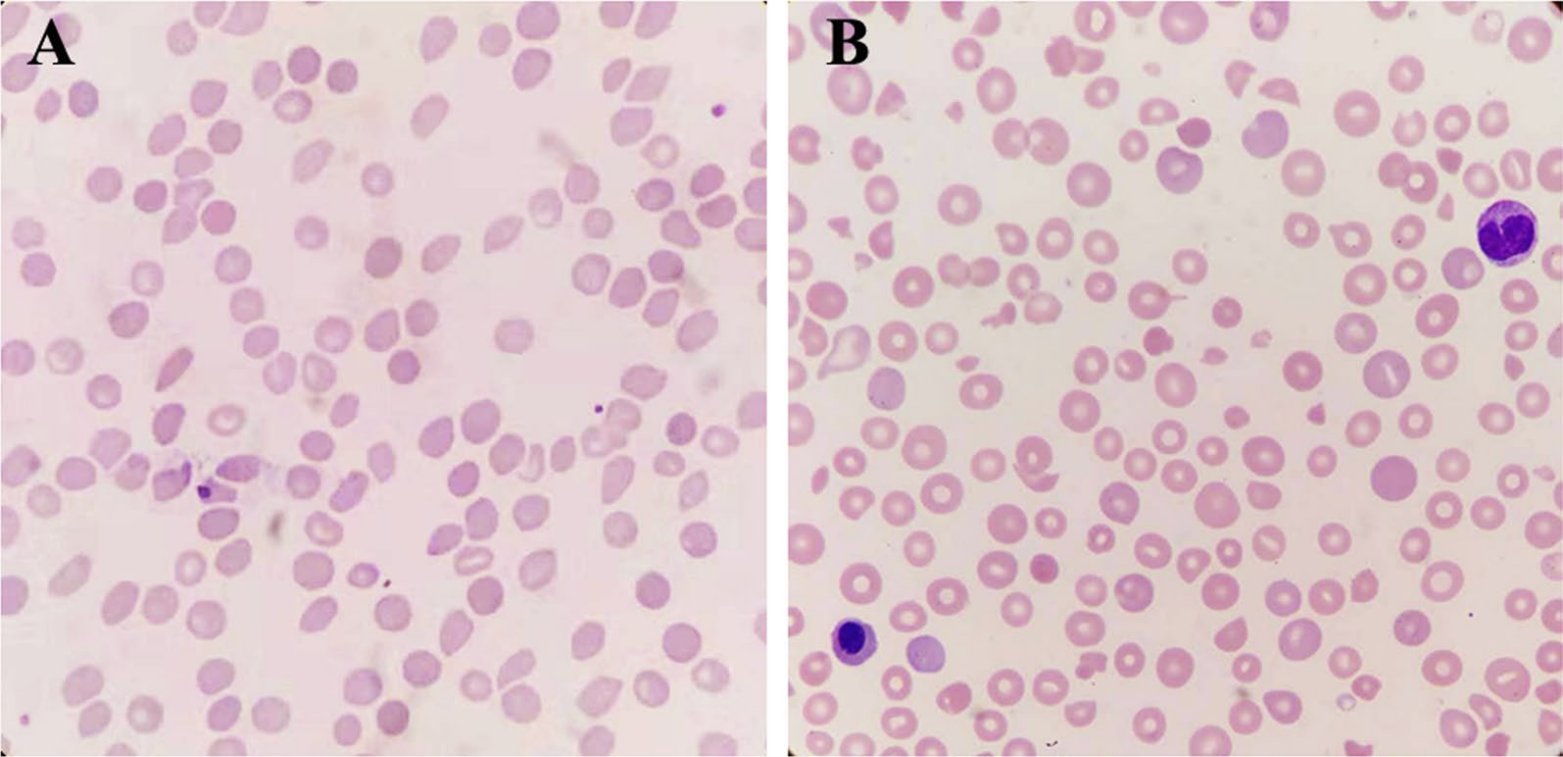
Peripheral blood smear showed microangiopathic hemolytic anemia with schistocytosis, **A** On day of admission, the smear had few schistocytes. **B** One week later, the smear showed an increased number of schistocytes that raised the question of microangiopathic hemolytic anemia

He remained asymptomatic but over 9 days, he experienced several episodes of headache, blurred vision and minor mental status changes, with fever high up to 38.5. Moreover, Peripheral smear showed an increased number of schistocytes (1.3%) (Fig. 1). PEX through a right femoral venous hemodialysis catheter was carried out daily immediately after the onset of neurologic abnormalities immediately even if ADAMTS-13 levels remained unknown given the high risk of morbidity and mortality of TTP within the first 24 h if plasma replacement therapy is not given [4]. However, because the shortage of serum, we collected 1000 mL serum, then added 500 mL volume of albumin. The PEX procedure resulted in a dramatic response with improvements. His neurologic abnormalities resolved immediately and did not recur.

**Fig. 2:**
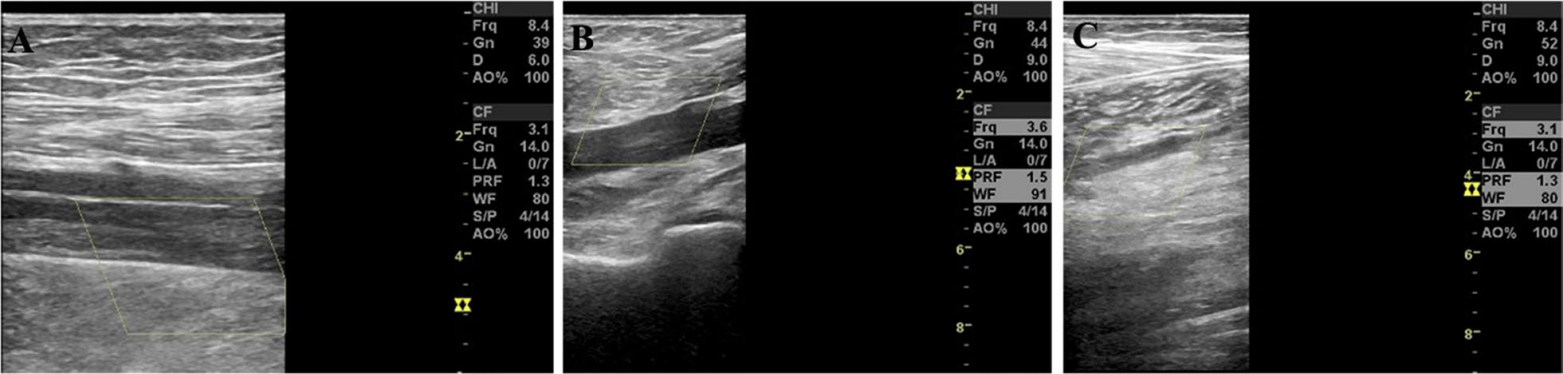
Color Doppler flow images revealing DVT involving the lower extremities. **A** DVT in the superficial femoral vein of the right leg. **B** DVT in the popliteal vein of the right leg. **C** DVT in the intermuscular vein of the right leg. No signals of blood flow were shown above. DVT, deep venous thrombosis

On the 3th day post PEX therapy, the PLT rise to 156 × 10^9^/L with the LDH level down to 478 U/L. Because complete response of PEX was defined by a full resolution of any neurological manifestations and platelet count recovery (> 150 × 10^9^/L) for at least two days based on previous studies and in accordance with international guidelines [5]. Therefore, we continued PEX therapy. On the 4^th^ day post PEX, continuous improvement was noted on the blood test, with platelets peaked to 195 × 10^9^/L and LDH down to 331 U/L. However, the patient presented with a sudden onset right leg swelling and pain. There were no associated signs or symptoms such as dyspnea or fever. The color Doppler ultrasound demonstrated evidence of DVT in the right lower extremity which showed total thrombosis of the right external iliac and femoral veins and nearly total thrombosis of the right popliteal vein (Fig. 2).

**Fig. 3:**
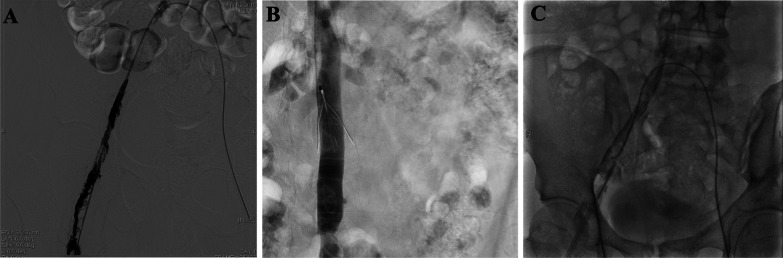
Venography of right iliac to popliteal vein. Angiography **A** before thrombectomy. **B** after thrombectomy with inserted perfusion catheter. **C** after urokinase thrombolysis

We consulted vascular surgeons. Based on their recommendation, the patient was implanted an inferior vena cava filter and underwent catheter thrombolysis and perfusion catheter insertion with continued administration of thrombolytic agent (Fig. 3). At the meantime, PEX session was sustained for another 2 days. The symptoms of DVT relieved markedly, and we shifted to an oral anticoagulant (rivaroxaban). After another twice PEX, he continued to remain asymptomatic, his hematological parameters stabilized with a platelet count of 200 × 10^9^/L at discharge and plasma D-dimer levels returned to normal. The patient is now under follow-up in the outpatient clinic and is undertaking rivaroxaban daily, while progressively tapering oral corticosteroids. In a yearly follow-up, there has been no anemia and the platelet count also remains normal to date.

## Discussion

Hemolysis in idiopathic TTP is mechanical and nonimmune mediated, thus Coombs testing is usually negative. Nowadays, autoimmune diseases caused acquired TTP. have been explored broadly, which caused vascular endothelial cell damage, release of a large number of vWF, lack of vWF-cp or inhibition of vWF-cp activity, leading to microaggregation of platelets and vWF-fibrinogen, vessel occlusion, and rapid reduction of platelets, and finally resulting in occurrence of TTP [6]. Coombs testing could be positive in that case, and usually ended with fatality in adult literature [7]. A variety of autoimmune disorders may develop several years after the recovery of TTP and such observations highlight the necessity of clinical surveillance [6], however, the guidelines are still missing.

The diagnosis of TTP requires clinical judgment in addition to measurement of ADAMTS13 activity [8]. Since rapid ADAMTS13 activity assessment is not available in routinely, leading to diagnostic wanderings with potentially severe consequences on prognosis by delaying therapeutic plasma exchange (TPE) in cases of diagnosis uncertainty. Our current practice continues to treat patients with PEX if they have clinical features of TTP with no alternative diagnosis, even if the ADAMTS13 activity is not available. However, we must balance the risks and benefits for PEX procedure at the first place. Common risks are as follows: hemorrhage or pneumothorax complicating the insertion of central venous catheter, thrombosis or sepsis attributed to central venous catheter, anaphylactic reaction to plasma and cardiac tamponade related to catheter insertion [9]. As in this case, the patient responded well with treatment of timely PEX and corticosteroids, however, femoral catheterization associated DVT occurred. It is most probably caused by endothelial damage secondary to intravenous catheters. Then, loss of physiological thromboresistance, leukocyte adhesion to damaged endothelium, complement consumption, abnormal vWF release and fragmentation, and increased vascular shear stress may then sustain and amplify the microangiopathic process. We hypothesis that tries to explain the complications of PEX considers that PEX removed autoantibodies and corrected PLT deficiency, resulting in thrombosis. Low-molecular-weight heparin thromboprophylaxis plus antiplatelet when the platelet count > 50 × 10^9^/L were suggested in clinical work [10]. Previous studies have shown that the frequency of mechanical complications is greater with femoral catheterization than with subclavian. And internal jugular catheterization [11]. However, no specific guidelines regarding catheterization pathway was made yet.

Although the PEX-based method is the recommended acquired TTP treatment worldwide [12], other options could be considered for treating recurring or refractory TTP cases, or when severe adverse effects related to PEX such as bleeding or thrombosis appear [13]. Since 2002, therapeutic interventions aiming to B-cell depletion and reduction of autoantibodies, with rituximab, appear very effective both as induction therapy for the initiation of remission, as well as maintenance therapy, some even advocate the use of rituximab as routine initial treatment together with PEX and corticosteroids [14]; however, the frequencies of severe neurologic abnormalities, exacerbations, and death have not changed, while the frequency of relapse has decreased [15]. Recently, treatment of acute episodes of TTP with increasing use of rituximab and the addition of new agents, such as caplacizumab [16] and recombinant ADAMTS13 showed to be more effective.

We anticipate that more effective treatment will improve the quality and duration of life for patients in remission from TTP. With more effective treatments, the need for PEX and the risks for complications from PEX may decrease.

## Conclusion

Catheter-related DVT under the setting of TTP or TTP recovery stage may be presented as a more fulminant form. Still, long-term follow-up of TTP patients is crucial to identify the occurrence of other autoimmune diseases, to control relapses and to evaluate psychophysical sequelae. Further development of both patients’ registries worldwide and innovative drugs is still needed to improve TTP management.

## Data Availability

All data generated during our study is included in this article.

## Abbreviations

TTP: Thrombotic thrombocytopenic purpura
vWF: Von Willebrand factor
ADAMTS13: A disintegrin and metalloproteinase with thrombospondin motifs 13
PEX: Plasma exchange
DAT: Direct antiglobulin test
AIC: Autoimmune cytopenias
DVT: Deep vein thrombosis
IVC: Inferior vena cava filter
TPE: Therapeutic plasma exchange
PT: Prothrombin time
APTT: Activated partial thromboplastin time
PLT: Platelets
LDH: Lactate dehydrogenase
